# Characterization and immunogenicity of a *Shigella flexneri* 2a O-antigen bioconjugate vaccine candidate

**DOI:** 10.1093/glycob/cwz044

**Published:** 2019-06-17

**Authors:** Neil Ravenscroft, Martin Braun, Joerg Schneider, Anita M Dreyer, Michael Wetter, Micha A Haeuptle, Stefan Kemmler, Michael Steffen, Dominique Sirena, Stefan Herwig, Paula Carranza, Claire Jones, Andrew J Pollard, Michael Wacker, Michael Kowarik

**Affiliations:** 2 Department of Chemistry, University of Cape Town, Rondebosch 7701, South Africa; 3 LimmaTech Biologics AG, Grabenstrasse 3, 8952 Schlieren, Switzerland; 4 Department of Paediatrics, University of Oxford, Oxford OX3 9DU, United Kingdom; 5 Wacker Biotech Consulting AG, Obere Hönggerstrasse 9a, 8103 Unterengstringen, Switzerland

**Keywords:** biosynthetic glycoconjugate vaccine, *Escherichia coli* glycosylation, functional antibodies, immunogenicity, *Shigella flexneri* 2a

## Abstract

Shigellosis remains a major cause of diarrheal disease in developing countries and causes substantial morbidity and mortality in children. Vaccination represents a promising preventive measure to fight the burden of the disease, but despite enormous efforts, an efficacious vaccine is not available to date. The use of an innovative biosynthetic *Escherichia coli* glycosylation system substantially simplifies the production of a multivalent conjugate vaccine to prevent shigellosis. This bioconjugation approach has been used to produce the *Shigella dysenteriae* type O1 conjugate that has been successfully tested in a phase I clinical study in humans. In this report, we describe a similar approach for the production of an additional serotype required for a broadly protective shigellosis vaccine candidate. The *Shigella flexneri* 2a O-polysaccharide is conjugated to introduced asparagine residues of the carrier protein exotoxin A (EPA) from *Pseudomonas aeruginosa* by co-expression with the PglB oligosaccharyltransferase. The bioconjugate was purified, characterized using physicochemical methods and subjected to preclinical evaluation in rats. The bioconjugate elicited functional antibodies as shown by a bactericidal assay for *S. flexneri* 2a. This study confirms the applicability of bioconjugation for the *S. flexneri* 2a O-antigen, which provides an intrinsic advantage over chemical conjugates due to the simplicity of a single production step and ease of characterization of the homogenous monomeric conjugate formed. In addition, it shows that bioconjugates are able to raise functional antibodies against the polysaccharide antigen.

## Introduction

*Shigella* is one of the five main pathogens causing diarrheal disease, resulting in 1 in 10 child deaths during their first 5 years of life (Kotloff et al. 2013; Platts-Mills et al. 2015). This results in about 800,000 fatalities annually, mainly in sub-Saharan Africa and South Asia (Liu et al. 2012). In addition, increasing antibiotic resistance means that the development of a vaccine preventing *Shigella* infections remains a high priority for the World Health Organization (WHO; Livio et al. 2014). *Shigella flexneri* is the major cause of shigellosis in endemic countries, accounting for up to 60% cases of shigellosis mainly in developing countries (Livio et al. 2014). *S. flexneri* is particularly prevalent in China, South and Southeast Asia, Egypt, Kenya and Peru, where up to 90% of cases are caused by *S. flexneri* subspecies (Cohen et al. 2001; Putnam et al. 2004; Wang et al. 2005; Brooks et al. 2006; von Seidlein et al. 2006; Zafar et al. 2009). The most prevalent *S. flexneri* O serotype is 2a, followed by 3a and 6. In addition to *S. flexneri*, *Shigella dysenteriae* O1 and *Shigella sonnei* are causes of shigellosis. Historically, *S. dysenteriae* O1 caused epidemics and pandemics during times of population upheaval; however, few cases have been reported since 1990 (Kotloff et al. 2017).

Several vaccine strategies have been exploited to prevent shigellosis, including live-attenuated vaccines, inactivated whole-cell vaccines, subcellular vaccines and purified subunit vaccines such as the O antigen conjugate vaccines (Walker 2015). However, the lack of a clear correlate of protection and relevant animal models has made the development of a *Shigella* vaccine very challenging (Levine et al. 2007; Phalipon et al. 2008; Kaminski and Oaks 2009).

Conjugate vaccines against bacterial infections caused by *Streptococcus pneumoniae*, *Neisseria meningitidis*, *Haemophilus influenzae* type b (Hib) (Vella and Pace 2015) and *Salmonella typhi* (Szu 2013) have been successfully licensed. All of these conjugates consist of capsular polysaccharides chemically conjugated to carrier proteins. In *Shigella*, the O antigen polysaccharide (O-PS) is the vaccine antigen with the best promise of conferring protection in humans. The O-PS is the O serotype specific component of the lipopolysaccharide (LPS) and can also be present as capsular polysaccharide (Caboni et al. 2015). The O-PS can be chemically conjugated to a carrier protein and tested as a conjugate vaccine (Robbins et al. 1992). An *S. sonnei* O-PS conjugated to EPA was shown to be 74% protective in a phase III efficacy trial in Israeli soldiers and was shown to be safe and immunogenic in children 4–7 and 1–4 years of age (Cohen et al. 1997; Passwell et al. 2001, 2009). However, protection was demonstrated only in children above 3 years of age (Passwell et al. 2003, 2009; Niyogi 2005). Epidemiological evidence suggests that O antigen-specific IgG correlates with protection from infection (Livio et al. 2014).

The conventional production of such a multivalent conjugated *Shigella* vaccine is highly complex. The extraction and chemical conjugation of these antigenic polysaccharides is more complicated than for capsular polysaccharides, as LPS requires laborious extraction and chemical detoxification. In addition, detoxification might interfere with the structural integrity of the O-PS, and industrial scale production has not yet been reported for *Shigella* O-antigen conjugates. The use of acid hydrolysis performed directly on bacterial cells in order to release the O-PS-core saccharides for *Salmonella* has been reported (Micoli et al. 2013); however, it still requires multiple steps to prepare the corresponding conjugate vaccine. Consequently, chemical conjugation presents challenges for the production of a multivalent *Shigella* O-PS vaccine.

An innovative *Escherichia coli* glycosylation technology overcomes these challenges and allows the development of a multivalent *Shigella* vaccine (Wacker et al. 2002; Fernandez and Wacker 2014). It is based on the combination of two recombinant pathways in *E. coli*. The ability of *E. coli* to synthesize heterologous polysaccharides on its carrier lipid undecaprenyl pyrophosphate (UPP) is combined with the general N-glycosylation system of *Campylobacter jejuni* that allows the conjugation of the polysaccharide to specific residues of any carrier protein (Feldman et al. 2005). Using this in vivo glycosylation system, the O-PS of *S. dysenteriae* O1 was expressed in *E. coli* and transferred from the activated carrier lipid to EPA. The bioconjugate (Sd1-EPA) was extracted from *E. coli*, purified and extensively characterized (Ravenscroft et al. 2016). When administered to humans, the bioconjugate was shown to be safe and immunogenic (Hatz et al. 2015).

In this work the technology is applied to the production of *S. flexneri* 2a bioconjugate, another essential serotype of a multivalent *Shigella* O-PS vaccine. We show the construction of a modified *E. coli* strain functionally expressing the lipid-linked *S. flexneri* type 2a PS, which serves as a substrate for the oligosaccharyltransferase PglB. PglB transfers the activated polysaccharide from the carrier lipid to specifically selected asparagine residues (N262 and N398) within the bacterial consensus sequence for N-glycosylation in the periplasmic carrier protein EPA, forming the Sf2a-EPA bioconjugate. The bioconjugate is purified from *E. coli* cells (Kämpf et al. 2015) and extensively characterized using the methodology described for Sd1-EPA (Ravenscroft et al. 2016). For the first time, a *Shigella* bioconjugate vaccine elicits antibodies in animals that are able to kill *S. flexneri* in the presence of complement in vitro. Following preclinical testing, the bioconjugate was subsequently shown to be safe and immunogenic in a phase I clinical trial (Riddle et al. 2016) thereby demonstrating this promising approach to the ultimate goal of developing a broad-spectrum *Shigella* vaccine (Chen and Kotloff 2016).

## Results

### Bioconjugate expression strain construction and confirmation of 2a O-polysaccharide expression

The standard W3110 *E. coli* host has been established as a suitable, biosafety level 1 host for bioconjugates production (Wacker et al. 2014; Ravenscroft et al. 2016). For the preparation of a Sf2a bioconjugation cell line, four chromosomal manipulations were required. Two of them (Δ*waaL* and Δ*araBAD*) were introduced to delete *E. coli* wt biosynthetic pathways that interfere with bioconjugation. Firstly, the gene encoding the O-antigen ligase WaaL was deleted. WaaL transfers UPP linked O-polysaccharide to lipid A core forming LPS. Knockout of this pathway ensures channeling of all UPP-linked O-PS to the glycosylation pathway. The second mutation was introduced to control arabinose concentrations during the fermentation process. Arabinose acts as a key inducer chemical for expression of the carrier protein for bioconjugation.

To genetically stabilize the Sf2a O-PS synthesis in *E. coli*, the gene clusters encoding the responsible biosynthetic machineries were stably integrated into the W3110 chromosome. It has been established that for the biosynthesis of the Sf2a specific O-PS, at least two pathways are required: one encoded in the *rfbY* cluster and one encoded in the *gtr* operon. The *rfbY* cluster sequence encodes genes responsible for synthesis of the O-PS backbone polymer. This backbone is branched by glucose residues to render it specific for Sf2a. The responsible enzymatic activity is originating from the prophage encoded *gtr* operon (Mavris et al. 1997) of which a homologous pathway exists in *E. coli* W3110 (Allison and Verma 2000).

**Fig. 1:**
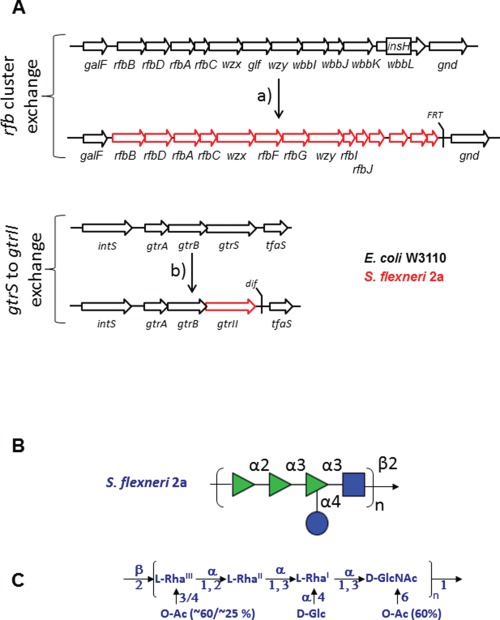
(**A**) Schematic description of the chromosomal modifications of the Sf2a-EPA production strain. Black framed arrows show W3110, red framed arrows indicate heterologous *S. flexneri* 2a open reading frames; gene annotations are shown below the arrows. The top panel shows the the O antigen cluster of *E. coli* W3110 before and after (a) exchange by the *rfbY* cluster from *S. flexneri* CCUG29416. Shown is the genomic locus spanning from *galF* to *gnd*; *InsH* indicates an insertion element in *wbbL* naturally present in W3110 and causes the O antigen negative phenotype of W3110. Flippase recognition target (FRT) indicates the “scar” in the genome due to the insertion/selection procedure using FRT recombination. Bottom panel: replacement of the serotype determining glycosyltransferase genes *gtrS* by *gtrII*. Shown is the genomic locus from *intS* to *tfaS*. *dif* indicates the scar in the genome due to the insertion/selection procedure using Xer recombination (Bloor and Cranenburgh 2006). (**B**) Schematic representation of the *S. flexneri* O-antigen biological repeat unit using the format suggested by the consortium for functional glycomics or (**C**) a classical representation also showing the non-stoichiometric O-sacetyl modifications of the carbohydrate backbone (Perepelov et al. 2009).

Therefore, to establish recombinant expression of the Sf2a O-PS, the *rfbY* gene cluster encoding the O-PS backbone from *S. flexneri* and the *gtrII* gene responsible for the Sf2a specific branching modification were integrated into the W3110 chromosome (Figure 1A). Both modifications were introduced by homologous recombination replacing the functional W3110-borne analogues. The *rfb*O16 cluster was replaced by *rfb*Y. The Sf2a specific, prophage-derived *gtrII* gene was inserted to replace its counterpart *gtrS* encoded in W3110 (Figure 1A). The desired genotypes were confirmed by colony PCR and genome sequencing of the modified loci. The functionality of the reconstituted Sf2a O-PS pathways was confirmed by use of *S. flexneri* specific serogroup typing sera (Levine et al. 2007). *E. coli* cells with a genomic copy of the heterologous *rfbY* O-PS gene cluster and expressing either the native periplasmic glucosyltransferase (GtrS) or the Sf2a specific glucosyltransferase (GtrII) were analyzed for their glycolipid content by western blotting of Proteinase K-treated whole cell extracts. Extracts from before and after manipulation using Type II specific typing sera showed the expected *S. flexneri* 2a specific reactivity only in the cells containing both chromosomal replacements (data not shown).

### Glycosylation of EPA with *S. flexneri* 2a O-PS and purification of the Sf2a-EPA glycoconjugate

**Fig. 2:**
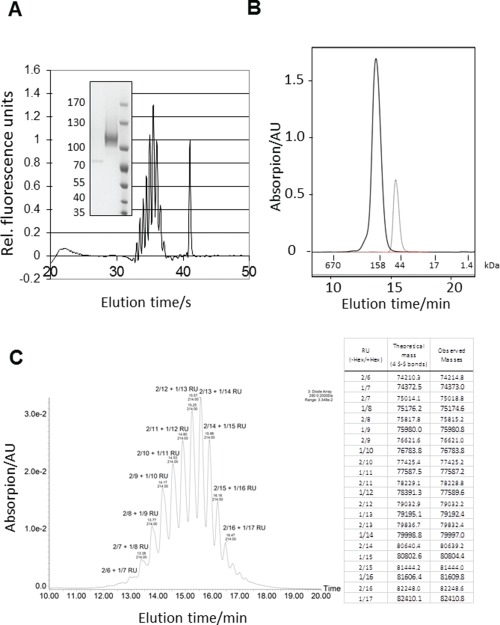
Analysis of Sf2a-EPA glycoconjugate. (**A**) Inset: Purified Sf2a-EPA (middle lane) was separated by SDS-PAGE and stained with Coomassie. Mono- and diglycosylated species were quantified using computational analysis of Coomassie signal intensities (not shown). Numbers of RUs were determined by linear regression using the relative migration distance of each rung of the ladder, representing a glycoform with a polysaccharide containing a defined number of RUs. Molecular weight ruler bands (right lane), corresponding molecular weights in kDa and unglycosylated EPA (left lane) are shown in the inset. Capillary gel electrophoresis electropherogram of purified Sf2a-EPA. The peak eluting at 41 s corresponds to the upper marker from the protein kit and corresponds to 230 kDa. This method enables high resolution of individual Sf2a-EPA glycoforms due to different number of RUs. The highest peak corresponds to ~14 RUs. (**B**) SE-HPLC chromatogram of purified Sf2a-EPA (solid) and unglycosylated EPA (dashed) as control for hydrodynamic size determination. Molecular weights of the gel filtration standard (Bio-Rad #151-1901) used for calculation are indicated at the bottom: 670 kDa: Thyroglobluline (bovine); 158 kDa: γ-globulin (bovine); 44 kDa: Ovalbumin (chicken); 17 kDa: Myoglobin (horse); 1.35 kDa: Vitamin B_12_. (**C**) Sf2E-EPA bioconjugate separation by hydrophilic interaction liquid chromatography (HILIC) followed by intact protein ESI-MS. Chromatographic separation of the purified bioconjugate preparation. For each peak two major glycoforms were identified by ESI MS, differing in mass by 1 hexose. The number of RUs lacking a hexoses is indicated before the number of RUs comprising a hexose after the forward slash. The “+” separates the two glycoforms identified in the same elution peak. The calculated and the observed glycoform masses are represented in tabular form to the right (see Figure **S3** for an overlay of all deconvoluted MS spectra from every peak).

To achieve conjugation of the Sf2a O-PS to the protein carrier EPA, plasmids encoding EPA and PglB were transformed into the W3110 strain containing all the genes encoding the biosynthetic machinery for Sf2a O-PS assembly as described above. Cells were grown at 37°C and expression of PglB and EPA was induced by the addition of isopropyl-β-D-thiogalactopyranoside (IPTG) and arabinose, respectively, and cells were grown overnight to stationary phase. Cells were harvested by tangential flow filtration (TFF), and bioconjugates were purified as described. Mainly glycosylated EPA was detected after final purification (above 100 kDa molecular size marker band, Figure 2A, inset), suggesting the covalent linkage of Sf2a repeating units (RUs) to the protein carrier. The main signal was constituted in a ladder-like pattern caused by the modally distributed O-PS attached to one glycosylation site in the EPA carrier protein between 100 and 130 kDa. Diglycosylated EPA was hardly detected as a very weak ladder signal with a similar distribution at 130 kDa. According to our quantification, Sf2a-EPA from the preparation shown features 92.7% monoglycosylated and 7.3% diglycosylated forms and an average RU length of 14. To obtain better resolution of the ladder-like pattern, capillary gel electrophoresis was performed (Figure 2A, main frame). Based on the peak spacing and extrapolation to non-glycosylated EPA, the number of RUs in the highest peak was determined to be 13, which is in good agreement with the SDS-PAGE data.

The Sf2a-EPA batch and the unglycosylated EPA reference standard were also analyzed by size exclusion high performance liquid chromatography (SE-HPLC; Figure 2B) calibrated using protein standards. The unglycosylated carrier protein EPA eluted with an apparent molecular weight of 66–68 kDa, which is in good agreement with the theoretical mass of 68.1 kDa. Analysis of Sf2a-EPA indicates the presence of mainly monoglycosylated forms eluting as a main peak at 13.8 min, while the fraction of diglycosylated conjugate is not sufficient for individual integration and is therefore only detectable as a shoulder of the peak at approximately 13.0 min. For the monoglycosylated species an apparent mass weight (MW) of 152 kDa was determined. The apparent MW of the complete conjugates was determined using protein gel filtration standards and this lead to an overestimation based on the hydrodynamic volume of the glycoconjugate. However, no significant traces of additional peaks due to aggregates, unglycosylated carrier protein, degradation products or impurities were detected. The anthrone assay was used to determine carbohydrate amounts. Typical yields from the process were 45 mg protein and 10 mg polysaccharide per liter fermentation broth, resulting in a polysaccharide to protein ratio of 22% (w/w). Free, unconjugated polysaccharide was not detected as measured by the free saccharide assay.

### Monosaccharide and polysaccharide compositional analysis of Sf2a-EPA confirms the *S. flexneri* 2a RU

As the *S. flexneri* O-antigen was recombinantly produced in *E. coli*, the attached saccharide was fully characterized to confirm that its structure was in agreement with the published RU (Perepelov et al. 2009). First, monosaccharide compositional analysis was performed as previously described (Ravenscroft et al. 2016). The identity of the peaks in the chromatogram of Sf2a-EPA (Figure **S1**A), assigned using monosaccharides standards, confirmed the presence of rhamnose (Rha), glucose (Glc) and N-acetylglucosamine (GlcNAc) in the RU as expected for the Sf2a O-PS (Figure 1B, C).

To obtain information about the glycan structure attached to the carrier protein, the Sf2a-EPA bioconjugate was subjected to hydrazinolysis. Chemically released glycans were labeled at their reducing end with the fluorophore 2-aminobenzamide (2-AB) and separated by normal phase HPLC. The resulting peaks of interest were collected and characterized by matrix-assisted laser desorption/ionization mass spectrometry (MALDI-MS/MS) analysis (Figure **S1**B). The masses found for the glycans eluting at 59.8 and 93.1 min matched the sodium adducts of the one and two RUs of the Sf2a glycan labeled with 2-AB, respectively. The MS/MS fragmentation ion series obtained for these precursor masses were consistent with the expected fragmentation pattern. The corresponding structures are schematically shown in Figure **S1**B above the individual peaks. The oligosaccharide 2-AB peaks characterized confirm the tetrasaccharide backbone of the O-PS and the presence of the branching hexose encoded by the exchanged GtrII glucosyltransferase. Additional peaks eluting at 39.9 and 82.2 min corresponded to glycan species labeled with 2-AB on the deoxyhexose. These were attributed to backbone PS hydrolysis of the Rha-GlcNAc linkage during the hydrazinolysis generating fragments with Rha residues at the reducing end. Thus, analysis of the constituent monosaccharides and polysaccharide sequence of the glycan component released from the carrier protein are consistent with the conclusion that the Sf2a O-PS RU Rha-Rha-Rha-GlcNAc with a branching Glc has been expressed in *E. coli*. The polysaccharides, eluting in the range of 140–165 min, consists of 10–16 RU.

**Fig. 3:**
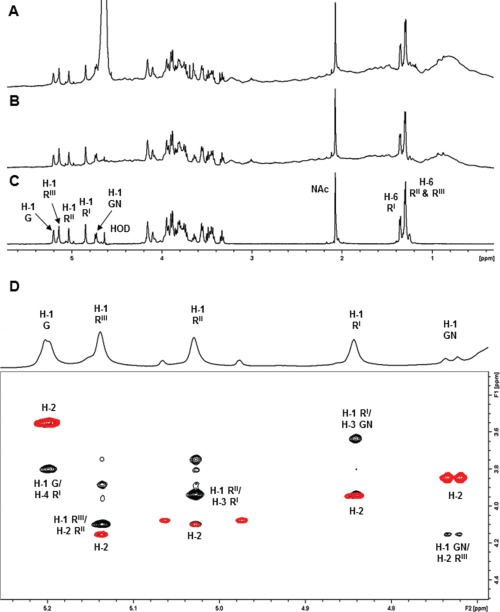
NMR spectra (600 MHz) of Sf2a-EPA and the derived glycopeptide recorded at 313 K. (**A**) ^1^H NMR spectrum of Sf2a-EPA; (**B**) 1D DOSY spectrum of Sf2a-EPA and (**C**) 1D DOSY spectrum of the Sf2a glycopeptide. Diagnostic anomeric and methyl signal are labeled (R = Rha, G = Glc and GN = GlcNAc). (**D**) ^1^H-^1^H NMR overlay of the anomeric region for Sf2a-EPA: COSY (red)/NOESY (black) recorded at 600 MHz (313 K). The major crosspeaks from H-1 are labeled (R = Rha, G = Glc and GN = GlcNAc).

To confirm that only the Sf2a glycan species are transferred by PglB to the carrier protein and to measure length and composition of the transferred glycans, intact glycoprotein analysis was performed. A purified bioconjugate was separated by hydrophilic interaction liquid chromatography (HILIC) and peaks in the elution (Figure 2C) were analyzed by electrospray ionization (ESI) MS. By deconvoluting the combined spectra of individual peaks, it was possible to identify masses related to expected masses originating from Sf2a-EPA. Each peak contained two major masses in agreement with a bioconjugate containing a specific Sf2a chain length lacking either one or two hexoses, likely related to the branched, GtrII-dependent glucose addition. Mass accuracy was high from 0.5 to 63 ppm for all confirmed glycoforms. Glycoforms ranged from 6 to 22 RU in length with a maximum intensity at 15 RUs. The deconvoluted masses of the peaks comprising EPA with 8–18 SF2 RUs are shown as an overlay in Figure **S2**. From the integrated peak areas of the ultra violet (UV) trace an average number of RU of 13.7 was calculated, which is in good agreement with the results from SDS-PAGE (14) and capillary gel electrophoresis (13) average RU determinations. This number of RUs is slightly higher compared to the results of the hydrazinolysis (10–16), which is expected because the polysaccharide chains are partially fragmented by the hydrazine treatment. The results observed give no indication about which glycosylation site is mainly used, but suggest that the majority of the glycoforms carry only one glycan chain. If both glycosites would be occupied a more complex glycoform pattern would be expected that likely could not be chromatographically resolved.

### Structural analysis of Sf2a-EPA by NMR spectroscopy

**Fig. 4:**
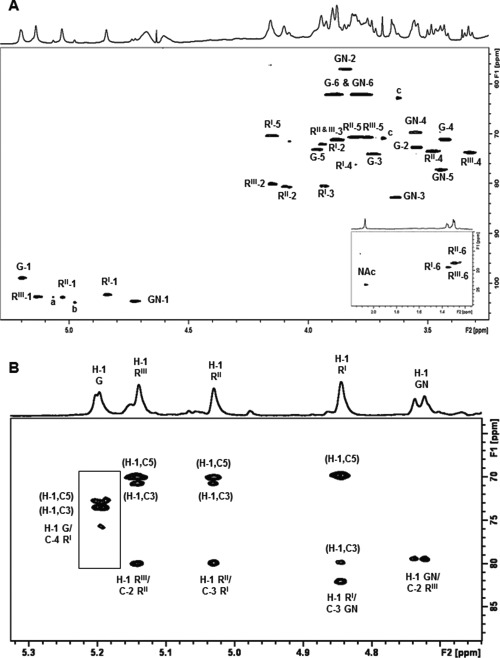
NMR spectra (600 MHz) of Sf2a-EPA and the derived glycopeptide recorded at 313 K. (**A**) Expansion of the HSQC spectrum of Sf2a-EPA, the crosspeaks from the methyl region of the spectrum are shown in the inset. Key pentasaccharide RU proton/carbon crosspeaks have been labeled according to the carbon atom of the corresponding residue (R = Rha, G = Glc and GN = GlcNAc). Small peaks due to anomeric signals from the non-reducing end disaccharide α-L-Rha*p*-(1→2)-α-L-Rha*p*-(1→ and buffer are labeled a–c, respectively. (**B**) Expansion of the HMBC spectrum of the Sf2a glycopeptide optimized for *J* = 8 Hz showing the anomeric H-1 correlations. The inset shows the crosspeaks for Glc obtained using a second HMBC experiment optimized for *J* = 6 Hz. Proton/carbon crosspeaks have been labeled according to the corresponding residue (R = Rha, G = Glc and GN = GlcNAc).

The ^1^H NMR spectrum of the Sf2a-EPA conjugate (Figure 3A) contained sharp signals due to the Sf2a saccharide superimposed on broad peaks of low intensity from the EPA protein. The 1D DOSY removed peaks from low MW components including the large water peak to clearly show the Sf2a signals (Figure 3B). The ^1^H NMR spectra showed the expected signals for the de-O-acetylated Sf2a pentasaccharide RU: four α- and one β- H-1 signals, ring signals, an N-acetyl signal at 2.07 ppm from β-GlcNAc and three methyl signals from H-6 of α-Rha (Perepelov et al. 2009). The five spin systems were elucidated using ^1^H-^1^H correlation experiments (COSY and TOCSY) that gave correlations from H-1 to H-6 for α-Glc and β-GlcNAc and H-1 to H-2 for the three α-Rha residues (Figure **S3**A). The remaining α-Rha proton assignments were made from correlations established from H-6 to H-2 (or H-1) for the three α-Rha residues (Figure **S3**B). The sequence of sugar residues followed from the transglycosidic correlations in the NOESY experiment: H-1 of α-Glc/H-4 of α-Rha^I^, H-1 of α-Rha^III^/H-2 of α-Rha^II^, H-1 of α-Rha^II^/H-3 of α-Rha^I^, H-1 of α-Rha^I^/H-3 of β-GlcNAc and H-1 of β-GlcNAc/H-2 of α-Rha^III^ (Figure 3D). All the HSQC crosspeaks for the Sf2a-EPA conjugate (Figure 4A) could be assigned from the proton assignments and are in agreement with literature values for the de-O-acetylated RU (Perepelov et al. 2009). The rapid transverse relaxation of the high MW bioconjugate gave poor 2D long-range correlation spectra and therefore additional NMR experiments were performed on the low MW Sf2a glycopeptide.

**Fig. 5:**
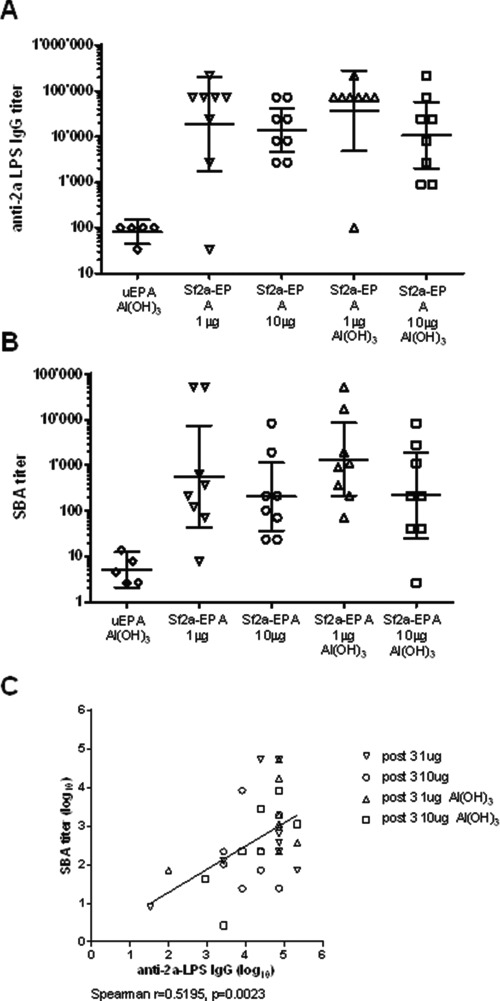
Immunogenicity in rats of Sf2a-EPA and serum bactericidal activity of corresponding sera. (**A**) Anti-Sf2a LPS IgG responses after vaccination with 1 and 10 μg PS doses with and without Alhydrogel® (Al (OH)_3_). Lines represent the GMT +/− 95% confidence interval. Mann–Whitney Exact *P*-values for comparison to the control group vaccinated with unglycosylated EPA (uEPA): 0.0163 (1 μg Sf2a-EPA), 0.0008 (10 μg Sf2a-EPA), 0.0039 (1 μg Sf2a-EPA, Al (OH)_3_) and 0.0016 (1 μg Sf2a-EPA Al (OH)_3_). (**B**) Serum bactericidal activity against *S. flexneri* 2a strain in post-immunization sera. Lines represent the GMT +/− 95% confidence interval. Mann–Whitney Exact *P*-values for comparison to the control group vaccinated with unglycosylated EPA (uEPA): 0.0039 (1 μg Sf2a-EPA), 0.0016 (10 μg Sf2a-EPA), 0.0016 (1 μg Sf2a-EPA, Al (OH)_3_) and 0.0135 (1 μg Sf2a-EPA Al (OH)_3_). (**C**) SBA titers are correlated with serum anti-Sf2a LPS IgG titers. Individual SBA titers plotted against anti-2a LPS IgG titers in 32 paired post-vaccination serum samples from all groups vaccinated with Sf2a-EPA.

The 1D DOSY spectrum of the Sf2a glycopeptide (Figure 3C) gave the same saccharide signals as the parent bioconjugate (Figure 3B). Full ^1^H and ^13^C NMR assignments were made using the same set of experiments applied to Sf2a-EPA aided by overlays with 1D TOCSY (200 ms), HSQC-TOCSY, HSQC-NOESY and HMBC experiments. The sequence and linkage positions of sugar residues indicated by glycosylation shifts and NOESY correlations was further corroborated by HMBC inter-residue correlations: H-1 of α-Glc to C-4 of α-Rha^I^, H-1 of α-Rha^III^ to C-2 of α-Rha^II^, H-1 of α-Rha^II^ to C-3 of α-Rha^I^ and H-1 of α-Rha^I^ to C-3 of β-GlcNAc (Figure 4B).

Additional small peaks at 4.97 and 5.07 ppm in the ^1^H NMR spectra of Sf2a-EPA and the corresponding glycopeptide (Figure 3) were attributed to anomeric protons from the non-reducing end RU. Examination of the small crosspeaks in the 2D correlation spectra showed that the spin systems for the H-1 signal at 4.97 ppm (C-1 at 103.8 ppm) and H-1 at 5.07 ppm (C-1 at 102.7 ppm) could be assigned to the non-reducing end disaccharide α-L-Rha*p*-(1→2)-α-L-Rha*p*-(1→, as reported for the SF2a core-1RU fragment isolated by acid hydrolysis (Kubler-Kielb et al. 2010). Thus, NMR analysis confirms the structure of the biosynthetically produced de-O-acetylated pentasaccharide RU of *S. flexneri* 2a as →2)-α-L-Rha*p*-(1→2)-α-L-Rha*p*-(1→3)-(α-D-Glc*p*-(1→4))-α-L-Rha*p*-(1→3)-α-D-Glc*p*NAc-(1→.

### Immunogenicity of Sf2a-EPA in rats and functionality of anti-polysaccharide antibodies

To assess immunogenicity and functionality of the Sf2a-EPA bioconjugate, a preclinical evaluation was performed. As there are no preclinical models available predictive for shigellosis in humans, we have used an animal model in which we had confidence to measure an immune response to assess Serum bactericidal assay (SBA) activity. Injection of Sprague–Dawley rats with Sf2a-EPA elicited Sf2a LPS-specific antibody responses (Figure 5A). Albeit individual responses were heterogenous, the Sf2a-LPS specific IgG geometric mean titers (GMT) in post vaccination sera of animals vaccinated with 1 μg and 10 μg polysaccharide doses (based on the Sf2a O-PS part of the conjugate) of Sf2a-EPA were significantly higher than in the group of animals vaccinated with unglycosylated EPA (*P* < 0.05). Both tested doses formulated with and without aluminum hydroxide elicited comparable anti-Sf2a LPS IgG titers.

Heterogeneity of the individual responses was not unexpected. It is known that the immune system of outbred rat strains react diversely to immunization. Furthermore, responses to polysaccharides have been seen to be more diverse between individuals compared to protein responses (van den Dobbelsteen et al. 2016).

To assess if vaccination with Sf2a-EPA elicits immune responses able to kill *S. flexneri* 2a bacteria, the vaccine induced antibodies were tested in a serum bactericidal assay. Neither pre-vaccination serum samples (data not shown) nor post vaccination samples from animals vaccinated with unglycosylated EPA showed bactericidal activity (Figure 5B). Serum samples from animals vaccinated with 1 μg and 10 μg dose of Sf2a-EPA with and without Alhydrogel®, showed significant bactericidal activity. Although the significance of the data is moderate, the bactericidal activity of the post vaccination sera correlated with their Sf2a-LPS specific IgG titers (Figure 5C). SBA is known to be affected by the difference in capacity for microbial opsonization between IgG subclasses and IgM. Thus, the moderate correlation may be due to different Ig subclass responses in individual animals, as well as intrinsic to the heterogeneity of the immune response in general (Figure 5C). In summary, these data confirm that Sf2a-EPA is able to raise a functional, bactericidal, antibody-dependent immune response in rats.

## Discussion

This study describes the production, characterization and preclinical testing of an O-PS bioconjugate vaccine candidate against infections caused by *S. flexneri* 2a. Licensed conjugate vaccines are formed from randomly activated or terminally activated bacterial saccharides that are conjugation directly or via a spacer to native or derivatized carrier proteins to form lattice or monomeric glycoconjugates. Both the substrates and the derived conjugate must be produced and purified under good manufacturing practice (GMP) conditions; the quality of each substrate or intermediate must be assessed and released before use in the conjugation reaction. This multi-step process requires control and characterization of each of the intermediates and the glycoconjugate drug substance produced as described in the WHO and Pharmacopoeia guidelines for Hib, meningococcal and pneumococcal vaccines (World Health Organization 2000, 2004, 2005, 2006). In contrast, bioconjugation is based on an enzymatic conjugation technology that has a single production step to yield a monomeric homogenous conjugate. This means a reduction not only of the number of GMP batches required to produce a single batch (from 3 to 1) but also a reduction of the number of analytical assays required to release the final product. In addition, because of their less complex structures (fewer and defined attachment sites) bioconjugates can be readily characterized, as recently described for a *S. dysenteriae* O1 (Sd1-EPA) bioconjugate vaccine (Ravenscroft et al. 2016). The production of Sf2a-EPA, the second and importantly indispensable component of a multivalent *Shigella* vaccine, demonstrates the feasibility of using the bioconjugation platform to develop multivalent LPS-based conjugate vaccines.

We achieved functional reconstitution of the *S. flexneri* 2a O-PS biosynthesis pathway, which includes a branching glucose by introducing the required genes from *S. flexneri* into *E. coli* resulting in a stable expression system that does not require antibiotic selection for vaccine expression. The glycan expression for Sf2a-EPA was further optimized compared to that described for Sd1-EPA and additionally required the introduction of a branching glucose (Sd1 has a linear RU). The O-PS expression pathway for Sd1-EPA was encoded on a plasmid containing all the genes for the O-antigen cluster. However, retention of the large plasmid during bacterial growth required antibiotic selection (Ravenscroft et al. 2016). For Sf2a-EPA, the gene clusters encoding the enzymes for the biosynthesis of the Sf2a PS were integrated into the *E. coli* host thereby allowing bioconjugate expression without the need for antibiotic selection. This substantially simplifies the bioconjugate production process and facilitates the development of a multivalent conjugate vaccine using this technology.

The attachment of the Sf2a-specific branching glucose to the O-PS backbone is encoded in the prophage derived *gtr* gene cluster. The last gene in the cluster, gtrII, encodes the glycosyltransferase GtrII responsible for the glucose transfer to its specific position in Sf2a. In *E. coli*, a homologous *gtr* system is present. The enzymes GtrA and GtrB, which covalently link the glucose to the carrier lipid and transport it to the periplasm, collaborate functionally with the third, specifying enzyme, the O16 periplasmic glucosyltranferase, encoded by the gene *gtrS*. Our data show that an exchange of the *E. coli* O16 specific glycosyltransferase gene *gtrS* by the Sf2a specific *gtrII* is sufficient for synthesis of the Sf2a specific polysaccharide branching modification and that the *E. coli* derived GtrA and GtrB enzymes can collaborate with GtrII from *S. flexneri* 2a. The presence of glucose in the RU was shown by sugar composition analysis, 2-AB analysis after hydrazinolysis of Sf2a-EPA and ESI-MS. Lastly, attachment of terminal α-Glc to C-4 of the 3,4-linked α-Rha^I^ of the Sf2a RU was demonstrated by detailed NMR characterization of Sf2a-EPA and the derived glycopeptide.

The native Sf2a O antigen RU contains non-stoichiometric O-acetylation; however, antibody binding and of synthetic oligosaccharides and preclinical testing of a synthetic Sf2a-tetanus toxoid conjugate vaccine performed in mice indicated that O-acetylation may not be important for immunogenicity and functionality (Phalipon et al. 2009; Gauthier et al. 2014). Our data are in agreement with these findings. The biosynthetic Sf2a-EPA was developed without O-acetylation; the absence of O-acetyl groups was confirmed by NMR spectroscopy. Rats immunized with Sf2a-EPA elicited IgG that is able to bind to *S. flexneri* 2a and that killed *S. flexneri* 2a cells in vitro in the presence of complement. Both immune measures were previously reported to be associated with protection against shigellosis (Cohen et al. 1988; Cohen et al. 1989; Robin et al. 1997; Shimanovich et al. 2017).

O-acetylation is also not required for functional glycoconjugate vaccines against Sf2a in humans. The Sf2a-EPA bioconjugate vaccine presented here has been tested in a clinical phase I study. It showed that the vaccine elicited functional antibodies as measured by SBA against living *S. flexneri* 2a bacteria (Riddle et al. 2016). Thus, the Sf2a-EPA bioconjugate described herein elicits functional antibodies and is a promising step toward the development of an efficacious multivalent *Shigella* bioconjugate vaccine.

The bioconjugation technology described herein simplifies the production of conjugate vaccines and the associated analytics. Unlike chemical conjugates prepared from LPS or by multiple steps of synthesis to form the antigen for conjugation, bioconjugates result from a single fermentation/processing step. The process is highly reproducible and the homogenous conjugate with defined polysaccharide antigen attachment sites is amenable to physicochemical characterization using simple and direct biochemical methods. The feasibility of producing a cost effective multivalent *Shigella* vaccine is challenging (van der Put et al. 2016). We will use the bioconjugation approach for *S. flexneri* 3a and 6, which together with *S. sonnei*, is also required for a broadly protective multivalent shigellosis vaccine (Livio et al. 2014). A clinical study to assess the efficacy of this Sf2a-EPA bioconjugate against experimental oral challenge is underway. Efficacy data in humans will be the next milestone for the development of a vaccine against one of the most devastating global enteric diseases.

## Materials and methods

### Bacterial strains, plasmids and growth conditions

*E. coli* and *S. flexneri* strains were grown in LB at 37°C. Kanamycin (Kan), 50 μg/mL; tetracycline (Tet), 20 μg/mL; spectinomycin (Sp), 80 μg/mL; chloramphenicol (Clm), 20 μg/mL; and ampicillin (Amp), 100 μg/mL were added to the media for selection as needed. *E. coli* DH5α (Life Technologies, Carlsbad, CA) was the host for cloning experiments. Plasmids pEXT21 and pBR322 (NEB, Beverly, MA) were used as cloning vectors. *E. coli* W3110 was from the Coli Genetic Stock Center, Yale University, New Haven, CT. Deletion of the *waaL* chromosomal gene in W3110 was performed as previously described (Ravenscroft et al. 2016).

The host strain for the production of Sf2a-EPA glycoconjugate was prepared by engineering of the chromosome of W3110 using different methods, resulting in the following genotype: *E. coli* W3110 Δ*rfbW3110*::*rfbCCUG29416* Δ*waaL* Δ*gtrS::gtrII* Δ*araBAD*. The complete rfb O antigen biosynthesis cluster sequence was from CCUG29416 (Culture collection of the University of Goetheborg, Sweden), and the gtrII gene (from strain 2457T) was obtained by gene synthesis (Genescript, Piscataway, NJ). A detailed description of the chromosomal engineering methods and confirmation procedures have been published (WO2014057109A1). The *S. flexneri 2a* strain CCUG29416 was used for extraction of LPS O-polysaccharide.

The detoxified carrier protein EPA protein and the PglB oligosaccharyl transferase were expressed as described previously (Ravenscroft et al. 2016) with slight modifications of the expression plasmids (Table **I**).

**Table I:** Strains and plasmids used in this study

Strain	Characteristic	Reference
DH5α	F- φ80*lac*Z∆M15 ∆(*lac*ZYA-*arg*F) U169 *deo*R *rec*A1 *end*A1 *hsd*R17 (r_k_-, m_k_+) *gal*^−^*pho*A *sup*E44 λ^−^*thi*^−^1 *gyr*A96 *rel*A1	Clontech Laboratories Inc., Mountain View, CA
W3110	*rph-I IN (rrnD-rrnE) 1*	Coli Genetic Stock Center, Yale University, New Haven, CT
*CCUG29416*	*Shigella flexneri CCUG29416 (Batch 911128); Serovar 2a; isolation 1991 from Human feces, Germany, Hamburg*	CCUG
Plasmid	Description	Reference
pEXT21	tac promoter expression vector; SpR	Dykxhoorn et al. (1996)
pEXT22	tac promoter expression vector; KanR	
p114	HA-tagged pglB cloned in pEXT21, IPTG inducible, SpR	Ihssen et al. (2010)
p150	Soluble periplasmic His6-tagged toxoid variant (L552V, DE553) of *Pseudomonas aeruginosa* EPA containing two glycosylation sequences cloned into pEC415, arabinose inducible; AmpR	Ravenscroft et al. (2016)
p970	Expression vector for HA-tag less, codon usage optimized PglB otherwise identical to p114, SpR	This study
p1198	In p150 the Amp resistance cassette was replaced by the one from pEXT22, and subsequently the his tag was removed by qick change mutagenesis (Genescript)	This study

### Production of Sf2a LPS and O-PS reference material

LPS was extracted from *S. flexneri* 2a strain CCUG29416 as previously described (Ravenscroft et al. 2016).

### Production and purification of glycosylated EPA (Sf2a-EPA)

To produce Sf2a-EPA, 1 mL of glycerol stock *E. coli* cells expressing the Sf2a O-PS as described above with plasmids p293 and p114 containing genes encoding EPA and PglB, respectively, were used. Biomass was produced in a stainless steel bioreactor by a fed-batch fermentation process using a complex medium under aerobic conditions as described (Kämpf et al. 2015). The culture was inoculated from an overnight culture and induced during the exponential growth phase with arabinose and IPTG. Biomass was harvested by TFF when product formation and biomass reached a maximum. Biomass was stored at −80°C.

Sf2a-EPA was extracted from the periplasm by osmotic shock and purified as described previously (van den Dobbelsteen et al. 2016). Fractions containing Sf2a-EPA were pooled and used for analysis.

### Degree of glycosylation and analytical SEC

Characterization of the degree of glycosylation was performed by an SDS-PAGE-based method to provide information about the polysaccharide chain length distribution within the different glycosylation forms (mono- and diglycosylated). The amount of mono- and diglycosylated species was determined by integration of the corresponding gel sections. Polysaccharide chain length was determined by counting the rungs of the ladder, each corresponding to a defined number of Sf2a RUs linked to EPA. SDS-PAGE was performed on NuPAGE 3–8% Tris-Acetate gels (Life Technologies #EA0375BOX) in NuPAGE Tris-Acetate buffer (Life Technologies #LA0041).

Capillary gel electrophoresis was performed on an 2100 Bioanalyzer instrument (Agilent: Agilent Technologies, Basel, Switzerland). Samples and the ladder were prepared and the chip was set up and run as described in the Agilent Protein 230 Kit Quick Start Guide.

Size exclusion chromatography (SEC) was performed on a TSKgel-G3000 SWxl column (TOSOH Bioscience) in 1 × PBS pH 7.5 and monitored by an UV detector at 215 nm. This method was further used to determine relative retention times and apparent molecular weights of the conjugates using appropriate gel filtration protein standards (Sigma-Aldrich, St Louis, MO, #MWGF1000; Bio-Rad (Cressier, Switzerland) #151–1901). Truncation or even complete loss of PS chains was monitored by apparent MW determination.

### Intact mass determination by mass spectrometry

An intact protein MS analysis was carried out following a HILIC-based separation on a glycoprotein column. The chromatographic separation of the glycoforms was performed using a Glycoprotein BEH Amide column (Waters (Milford, MA); 300 Å, 1.7 μm, 2.1 mm × 150 mm) on a Waters Acquity UPLC system. The separation of the bioconjugate was achieved with a gradient starting at 25% of 0.1% trifluoroacetic acid (TFA) in water (buffer A) and 75% of 0.1% TFA in in acetonitrile (buffer B) going to 55% buffer A and 45% buffer B in 30 min at a flow rate of 0.2 mL/min. Detection was performed using an UV detector at 215/280. The UPLC was directly connected to a Waters Synapt G2Si Q-TOF instrument for mass determination run in resolution mode. Masses were acquired in the positive ion mode between m/z 500 and 4500 with conditions adapted for intact protein analysis. Lock mass correction was applied. MS spectra were deconvoluted with the MassLynx MaxEnt 1 algorithm (Waters) to determine the average intact masses of the glycoproteins injected. For the deconvolution, spectra belonging to an individual peak were combined, smoothed and background subtracted and the deconvolution carried out individually for each of these peaks.

### Glycan and protein content for concentration and sugar to protein ratio determination measurements

The anthrone method was performed as described (Ravenscroft et al. 2016). A standard curve was generated using a mixture of the monosaccharides as present in the Sf2a polysaccharide in the expected molar ratios, including the branching glucose (D-GlcNAc:D-Glc:L-Rha = 1:1:3).

The total protein content for the Sf2a-EPA bioconjugate was determined by the micro bicinchinonic acid (μBCA) assay. The analytical procedure is performed using BSA as a reference standard and the μBCA Protein Assay Kit (Thermo Fisher Scientific,Waltham, MA, no. 23235). The glycan to protein weight ratio was calculated based on the protein content determined by μBCA assay and the glycan content determined by Anthrone assay (represented as % w/w).

### Monosaccharide compositional analysis, free saccharide analysis and hydrazinolysis

For confirmation of the monosaccharide identity, 10 μg polysaccharide of Sf2a-EPA was treated with TFA and then derivatized with 1-phenyl-3-methyl-2-pyrazoline-5-one (PMP) and analyzed by C18 reverse phase HPLC with detection by UV at 250 nm (Ravenscroft et al. 2016).

The amount of free polysaccharide was determined by a quantitative monosaccharide composition analysis using PMP. Briefly, the bioconjugate was removed by chromatography on a C4-cartridge (Macherey & Nagel, Düren, Germany). The free oligosaccharides in the resulting flow through fraction were hydrolyzed by harsh TFA treatment (6 h at 99°C in 3 M TFA). The resulting monosaccharides were labeled with PMP and separated as described above. The amount of the monosaccharide GlcNAc, mono-molar present in the Sf2a RU, was normalized using the internal standard galactose (Gal) and quantified by integration. The amount of free polysaccharide was calculated relative to the signal obtained from an identical bioconjugate sample processed without prior C4-cartridge treatment.

For hydrazinolysis, Sf2a-EPA corresponding to 1 mg protein was treated using the Ludger Liberate^TM^ Hydrazinolysis Glycan Release Kit (Ludger Ltd, Oxfordshire UK) and labeled by 2-AB as previously described (Ravenscroft et al. 2016). The 2AB-labeled saccharides were separated by normal phase HPLC, and their monosaccharide sequence was analyzed by MALDI-MS/MS as described (Wetter et al. 2013).

### NMR spectroscopy

Sf2a bioconjugate and glycopeptide samples (~1 mg of polysaccharide) were lyophilized and exchanged twice with 99.9% deuterium oxide (Sigma Aldrich), then dissolved in 600 μL of D_2_O and introduced into a 5 mm NMR tube for data acquisition. The 1D ^1^H, DOSY and TOCSY and 2D, COSY, TOCSY, NOESY, HSQC, HMBC and hybrid HSQC-TOCSY and HSQC-NOESY NMR spectra were obtained using a Bruker Avance III 600 MHz NMR spectrometer equipped with a BBO Prodigy cryoprobe and processed using standard Bruker software (Topspin 3.2). The 2D ^1^H-^1^H spectra were recorded with 50% non-uniform sampling (NUS) and 4k × 512 data points. The 2D ^1^H-^13^C spectra were recorded with 25% NUS and 4k × 512 data points for HSQC, HSQC-TOCSY and HSQC-NOESY and 4k × 256 data points for HMBC. The NUS multidimensional NMR data were processed using Multi-Dimensional Decomposition developed previously (Orekhov et al. 2003) and implemented in TopSpin. The probe temperature was set at 313 K. The 2D TOCSY experiments were performed using mixing times of 120 or 180 ms and the 1D variants using mixing times ranging from 30 to 200 ms. The NOESY spectra were recorded using a mixing time of 300 ms. The HSQC experiment was optimized for *J* = 145 Hz, and the HMBC experiments optimized for *J* = 6 and 8 Hz. HSQC-TOCSY and HSQC-NOESY NMR spectra were recorded using mixing times of 120 and 300 ms, respectively. Spectra were referenced to H-1/C-1 of α-Glc (^1^H signal at 5.20 ppm and ^13^C signal at 98.9 ppm; Perepelov et al. 2009). Sf2a glycopeptides were prepared by Pronase E (Sigma-Aldrich) digestion of Sf2a-EPA and subsequent purification on ENVI Carb (Supelco, München, Germany) and PD10 (GE Healthcare Life Sciences, München, Germany) columns.

### In vivo studies

Rats were maintained, immunized and bled at the animal facilities of Eurogentec SA (Liege Science Park, Seraing, Belgium) and studies were conducted according to the guidelines of the Federation of European Laboratory Animal Science Associations and UK Home Office Animals Scientific Procedures Act. In our experience immunization with bioconjugates induced a more consistent LPS-specific IgG response in rats compared to mice (data not shown).

Nine-week-old female Sprague Dawley rats (Janvier Labs, France) were injected intra-muscularly three times every 2 weeks with a 1 μg and 10 μg polysaccharide dose (corresponding to a 5.17 μg and 51.7 μg protein dose) of Sf2a-EPA with or without aluminium hydroxide gel (Alhydrogel®, Brenntag Biosector A/S, Frederikssund, Denmark). The Al^3+^ concentration was 0.02%. Control animals received injections with 51.7 μg of unglycosylated EPA.

### ELISA

Serum IgG titers against *S. flexneri* 2a LPS were measured by ELISA before vaccination and 14 days after the last injection. In brief, equal volumes of purified *S. flexneri* 2a LPS at 1 mg/mL and methylated bovine serum albumin (Sigma-Aldrich) at 1 mg/mL were mixed dropwise, diluted in PBS to a final concentration of 5 μg/mL LPS and coated on 96-well microtiter plates (MaxiSorp™, Nunc-Immuno™, Thermo Fisher Scientific) overnight at 4°C. After each incubation, plates were washed with PBS containing 0.05% Tween® 20 (PBST). Plates were blocked for 2 h at room temperature with 5% skimmed milk powder in PBST. Afterwards, plates were incubated for 1 h at room temperature with 3-fold serial dilutions of serum in PBST containing 2.5% skimmed milk powder (PBSTM). Specific IgG were detected with horseradish peroxidase-conjugated goat anti-rat IgG antibodies (Sigma-Aldrich) diluted in PBSTM and 3,3′,5,5′ tetramentylbenzidine substrate (1-Step™ Ultra TMB-ELISA, Thermo Fisher Scientific). The reaction was stopped with 2M sulfuric acid and the optical density was measured at 450 nm.

Antibody levels were expressed as endpoint titer, which corresponds to the highest dilution above the cut-off value corresponding to the mean + 3 times the standard deviation of the ODs of pre-immune sera serial dilutions. A titer of 100/3 = 33.3 was assigned to samples for which the lowest tested dilution (1:100) had an OD above the cut-off. Data analysis and graphical presentation of the results was performed using Microsoft Excel and GraphPad Prism software. Endpoint titers were compared between groups using the Mann–Whitney test.

### SBA

Log-phase grown *S. flexneri* 2a (strain 8519) were harvested, adjusted to a concentration of 0.1 OD_600_ and further diluted 1:5,000 in buffer (HBSS +0.5% BSA). The assay mixture was prepared in 96-well microtiter plates by combining 20 μL 3-fold serially diluted heat inactivated test serum and 10 μL bacterial suspension. After incubation at 37°C for 60 min with shaking, 10 μL of rabbit complement source was added to the wells (final assay concentration of 25%). Serum samples were tested and plated in duplicate. Controls were included, represented by test sera at lowest dilution with heat inactivated complement (Complement Independent Control), active complement without test serum (Complement Control) and heat inactivated complement without test serum (Viable Cell Counts). Plates were incubated at 37°C for 60–75 min with shaking and 10 μL from each well were dotted onto pre-labeled TSA plates using the tilt method. Colonies were counted after 16–18 h incubation at 37°C with 5% CO_2_ and SBA titers were calculated as the reciprocal serum dilution necessary to obtain 50% reduction in relation to the growth of bacteria obtained in the Viable Cell Count controls. A titer of 8/3 = 2.67 was assigned to samples with less than 50% killing for the lowest tested dilution (1:8). Results represent the geometric means for two to three separate experiments per sample. Data analysis and graphical presentation of the results is performed using Microsoft Excel and GraphPad Prism software. Endpoint titers were compared between groups using the Mann–Whitney test.

## Funding

South African National Research Foundation (Grant 86038, toward equipment funding); GlycoVaxyn AG; LimmaTech Biologics AG; GlaxoSmithKline Biologicals SA; Wellcome Trust (Strategic Translation Award 100527 assigned to Glycovaxyn AG and transferred to GlaxoSmithKline Biologicals SA in 2016).

## Conflict of interest statement

The employees of LimmaTech Biologics AG are receiving salary from their employer and may own shares of the company. The bioconjugation technology is owned by GlaxoSmithKline Biologicals SA and is protected by several patents.

## Supplementary Material

MS_Supp_data_rev_cwz044

## Abbreviations

2-AB: 2-aminobenzamide
Al (OH)_3_: Alhydrogel® adjuvans
Amp: ampicillin
Clm: chloramphenicol
COSY: Correlation Spectroscopy
DOSY: Diffusion Ordered Spectroscopy
EPA: Exotoxin A of *Pseudomonas aeruginosa*
ESI: electrospray ionization
Gal: galactose
Glc: glucose
GlcNAc: N-acetylglucosamine
GMP: good manufacturing practice
GMT: geometric mean titers
HBSS: Hank's balanced salt solution
Hib: *Haemophilus influenzae* type b
HILIC: hydrophilic interaction liquid chromatography
HMBC: Heteronuclear Multiple Bond Correlation
HSQC: Heteronuclear Single Quantum Correlation
IgG: immunoglobulin G
IgM: immunoglobulin M
IPTG: isopropyl-ß-D-thiogalactopyranoside
Kan: kanamycin
LB: Luria-Bertani
LPS: lipopolysaccharide
MALDI-MS/MS: 2 dimesional matrix-assisted laser desorption ionization mass spectrometry
NMR: Nuclear Magentic Resonance
NOESY: Nuclear Overhauser Effect Spectroscopy
NUS: non-uniform sampling
O-PS: O-polysaccharide
PCR: polymerase chain reaction
PMP: 1-phenyl-3-methyl-2-pyrazoline-5-one
Rha: rhamnose
RUs: repeating units
SBA: serum bactericidal assay
Sd1: O-antigen of *Shigella dysenteriae* type 1
Sd1-EPA: S. *dysenteriae* type 1 bioconjugate vaccine
SEC: size exclusion chromatography
SE-HPLC: size exclusion high performance liquid chromatography
Sf2a: O-antigen of *Shigella flexneri* type 2a
Sf2a-EPA: *S. flexneri* type 2a EPA bioconjugate
Sp: spectinomycin
Tet: tetracycline
TFA: trifluoroacetic acid
TFF: tangential flow filtration
TOCSY: Total Correlation Spectroscopy
uEPA: unglycosylated EPA
UPP: undecaprenyl pyrophosphate
WHO: World Health Organization
