# Supplementary material for: Characterization and immunogenicity of a Shigella flexneri 2a O-antigen bioconjugate vaccine candidate
Source: Glycobiology. 2019 Jun 17;29(9):669–80. doi: 10.1093/glycob/cwz044 (PMC6704370; doi:10.1093/glycob/cwz044)
Supplement: MS_Supp_data_rev_cwz044 [file ms_supp_data_rev_cwz044.docx]

**Supplementary information to**

**GlcNAc**

**Rha**

**Glc**

**min**

**20**

**24**

**28**

**32**

**36**

**40**

**44**

**48**

**mAU**

**0**

**20**

**40**

**60**

**80**

**100**

**120**

mAU

-10

0

10

20

30

40

50

60

70

80

90

100

120

**23.9**

**34.2**

**43.9**

**Characterization and immunogenicity of a *Shigella flexneri* 2a O-antigen bioconjugate vaccine candidate**

**Neil Ravenscroft^2^, Martin Braun^3^, Joerg Schneider^3^, Anita Dreyer^3^, Michael Wetter^3, 6^, Micha Haeuptle^3, 7^, Stefan Kemmler^3^, Michael Steffen^3^, Dominique Sirena^3^, Stefan Herwig^3^, Paula Carranza^3^, Claire Jones^4^, Andrew Pollard^4^, Michael Wacker^3, 5^, Michael Kowarik^1, 3^**

^2^Department of Chemistry, University of Cape Town, Rondebosch 7701, South Africa, ^3^LimmaTech Biologics AG, Grabenstrasse 3, 8952 Schlieren, Switzerland, ^4^Department of Paediatrics, University of Oxford, Oxford OX3 9DU, United Kingdom and ^5^Wacker Biotech Consulting AG, Obere Hönggerstrasse 9a, 8103 Unterengstringen, Switzerland

^6^Current address: Institute of Microbiology, ETH Zurich, Zürich, Switzerland

^7^Current address: Molecular Partners AG, Schlieren, Switzerland

^1^To whom correspondence should be addressed: Tel: +41447338580; Fax: +41447338576; e-mail: michael.kowarik@lmtbio.com

Key words: biosynthetic glycoconjugate vaccine / *E. coli* glycosylation /*Shigella flexneri* 2a / immunogenicity / functional antibodies

Running title: Characterization of a *Shigella* 2a vaccine candidate

**Figure S1**: Analysis of the polysaccharide antigen released from Sf2a-EPA bioconjugates. Purified Sf2a-EPA was analyzed for monosaccharide composition by hydrolysis followed by PMP labelling. (A) Arrows indicate the elution times of the monosaccharide standards treated in the same way as the bioconjugate, and detected at 250 nm. (B). Normal phase HPLC elution chromatogram of Sf2a-EPA bioconjugate after hydrazinolysis subsequent2-AB labelling. Main peaks were collected and analysed by MALDI-MS/MS. Resulting fragmentation ion series were interpreted as monosaccharide sequences and corresponding polysaccharide structures are indicated on top of the respective peaks. Triangles represent deoxyhexoses, squares N-aceylhexosamines, circles hexoses (according to the CFG nomenclature).

**Figure S2:** The deconvoluted spectra of the individual elution peaks were overlayed for visualization. Each peak contained two main glycoprotein species, indicated by the same color. These corresponded to species with the same number of repeating units with either one or two repeat units lacking a hexose.

**Figure S3**: The 2D ^1^H-^1^H overlay for Sf2a-EPA: COSY (red)/ TOCSY (black) recorded at 600 MHz (313 K). (A) The major crosspeaks from H-1 are labelled; (B) the major crosspeaks from H-6 of α-Rha^I^, α-Rha^II^ and α-Rha^III^ are labelled. (R = Rha, G = Glc and GN = GlcNAc).
